# Fibronectin and Periostin as Prognostic Markers in Ovarian Cancer

**DOI:** 10.3390/cells9010149

**Published:** 2020-01-08

**Authors:** Katarzyna Aleksandra Kujawa, Ewa Zembala-Nożyńska, Alexander Jorge Cortez, Tomasz Kujawa, Jolanta Kupryjańczyk, Katarzyna Marta Lisowska

**Affiliations:** 1 Center for Translational Research and Molecular Biology of Cancer; Maria Skłodowska-Curie National Research Institute of Oncology, Gliwice Branch, 44-100 Gliwice, Poland; katarzyna.kujawa@io.gliwice.pl (K.A.K.);; 2 Tumor Pathology Department, Maria Skłodowska-Curie National Research Institute of Oncology, Gliwice Branch, 44-100 Gliwice, Poland; 3 Data Mining Division, Faculty of Automatic Control, Electronics and Computer Science, Silesian University of Technology, 44-100 Gliwice, Poland; 4 Department of Pathology and Laboratory Diagnostics, Maria Skłodowska-Curie National Research Institute of Oncology, 02-781 Warsaw, Poland

**Keywords:** high-grade serous ovarian cancer, fibronectin, periostin, prognostic biomarkers, immunohistochemistry, tumor stroma

## Abstract

Previously, based on a DNA microarray experiment, we identified a 96-gene prognostic signature associated with the shorter survival of ovarian cancer patients. We hypothesized that some differentially expressed protein-coding genes from this signature could potentially serve as prognostic markers. The present study was aimed to validate two proteins, namely fibronectin (FN1) and periostin (POSTN), in the independent set of ovarian cancer samples. Both proteins are mainly known as extracellular matrix proteins with many important functions in physiology. However, there are also indications that they are implicated in cancer, including ovarian cancer. The expression of these proteins was immunohistochemically analyzed in 108 surgical samples of advanced ovarian cancer (majority: high-grade serous) and additionally on tissue arrays representing different stages of the progression of ovarian and fallopian tube epithelial tumors, from normal epithelia, through benign tumors, to adenocarcinomas of different stages. The correlation with clinical, pathological, and molecular features was evaluated. Kaplan–Meier survival analysis and Cox-proportional hazards models were used to estimate the correlation of the expression levels these proteins with survival. We observed that the higher expression of fibronectin in the tumor stroma was highly associated with shorter overall survival (OS) (Kaplan–Meier analysis, log-rank test *p* = 0.003). Periostin was also associated with shorter OS (*p* = 0.04). When we analyzed the combined score, calculated by adding together individual scores for stromal fibronectin and periostin expression, Cox regression demonstrated that this joint FN1&POSTN score was an independent prognostic factor for OS (HR = 2.16; 95% CI: 1.02–4.60; *p* = 0.044). The expression of fibronectin and periostin was also associated with the source of ovarian tumor sample: metastases showed higher expression of these proteins than primary tumor samples (χ^2^ test, *p* = 0.024 and *p* = 0.032). Elevated expression of fibronectin and periostin was also more common in fallopian cancers than in ovarian cancers. Our results support some previous observations that fibronectin and periostin have a prognostic significance in ovarian cancer. In addition, we propose the joint FN1&POSTN score as an independent prognostic factor for OS. Based on our results, it may also be speculated that these proteins are related to tumor progression and/or may indicate fallopian–epithelial origin of the tumor.

## 1. Introduction

Ovarian cancer is a highly heterogeneous disease, and this phenomenon is probably due to the distinct cellular origin of its individual histological types. Recent findings suggest that clear cell and endometrioid ovarian cancers originate from endometriosis, while serous ovarian cancers may originate either from the ovarian surface or fallopian tube epithelium [1,2,3]. The heterogeneity of ovarian cancer is also legible at the molecular level. We have shown previously, in our microarray study, that the histological type of the tumor is the major source of variability in the gene expression pattern [4,5]. The only exception to this concerned serous and undifferentiated tumors, which had near-identical molecular profiles. We concluded that gene expression studies should either be performed on histologically homogenous groups of cancer samples or, alternatively, the results of such studies must be filtered appropriately so as to exclude genes related with histological differences. Otherwise, huge differences in gene expression resulting from histological heterogeneity can obscure other, more subtle differences related to other features of interest. However, even when analyzing a homogenous group of serous/undifferentiated ovarian cancers, we found that they were not uniform at the molecular level. In fact, we could distinguish two subtypes of cancers with distinct gene expression patterns. Moreover, these subtypes were associated with differential survival: patients with higher expression of certain group of genes had shorter overall survival than those with lower expression of the same group of genes [5]. Our prognostic signature consisted mainly of genes related to extracellular matrix (ECM) structure and function. The underlying cellular adhesion and motility processes are known to be involved in the acquisition of invasive and metastatic phenotype by cancer cells. The present study was aimed to validate, using immunohistochemistry, selected genes from this signature, on the independent set of ovarian cancer samples. For this analysis, we have chosen two genes (proteins) associated with ECM: *FN1* (fibronectin) and *POSTN* (periostin). Both proteins have long been known mainly for their physiological functions, while later studies indicated that they are also implicated in cancer.

Cellular fibronectin has many functions in physiology, related with cell adhesion, growth, migration, and differentiation, playing important role e.g., in embryonic development and wound healing; reviewed in: [6,7,8]. In addition, fibronectin expression has been observed in several cancers; reviewed in: [9,10]. Functional studies demonstrate that fibronectin stimulates ovarian cancer cells proliferation and promotes metastasis by regulating ovarian cancer cells adhesion and invasion [11], reviewed in: [12]. Periostin was initially regarded merely as a structural component of connective tissues such as periodontal ligament, periosteum, fascia of skeletal muscles, and cardiac valve [13,14,15]. However, periostin is also overexpressed in different cancers, including ovarian; reviewed in: [16]. Periostin was shown to regulate ovarian cancer cells’ adhesion and motility [17,18], as reviewed in [12]. Moreover, some studies indicate that periostin, together with several other ECM proteins, is associated with drug resistance in ovarian cancer cell lines [19,20,21]. Both fibronectin [22,23] and periostin [24,25] have been suggested to be related with the survival of ovarian cancer patients.

Our results support previous indications that fibronectin and periostin are associated with a shorter survival of patients. In addition, we have proposed the joint FN1&POSTN score as an independent prognostic factor for OS. We also analyzed, using tissue arrays, whether these proteins show differential expression in different stages and distinct histological types of ovarian cancer, as well as in healthy and benign ovarian tissues. As it is postulated that serous cancers may have either ovarian or fallopian origin, we used arrays containing samples of both organs, including normal tissue, inflammatory states, benign and borderline tumors, and cancer. These experiments showed that elevated expression of fibronectin and periostin was more common in fallopian than in ovarian cancers. The elevated expression of fibronectin and periostin was also associated with the source of ovarian tumor sample: more samples with a higher expression of these proteins were among samples derived from omental metastatic disease than among other samples.

## 2. Materials and Methods

### 2.1. Tissue Arrays

We used OV1005a (US Biomax, Inc., Derwood, MD, USA) tissue arrays labeled as “ovary disease spectrum (ovarian cancer progression) tissue array”, containing 27 cases of serous adenocarcinoma, 3 mucinous adenocarcinomas, 10 endometrioid adenocarcinomas, 5 transitional cell carcinomas, 10 metastatic ovarian carcinomas, 25 ovary adenomas, 17 adjacent normal ovarian tissue, and 3 normal ovarian tissues, with a single core per case; total 100 cases/100 cores. In addition, we used UTE601 (US Biomax, Inc., Derwood, USA) arrays, described as “fallopian tube disease spectrum (fallopian tube cancer progression)”, containing 10 cases of each adenocarcinoma and inflammation, one tubal hyperplasia, 4 adjacent normal tissue and 5 normal tissue, duplicated cores per case. Tissue arrays were analyzed by immunohistochemistry. For preliminary test of IHC reaction parameters we used test arrays T112b described as “ovary cancer tissue array, with normal tissue control, including TNM, clinical stage and pathology grade” containing 12 cases/24 cores and UTE601 described as “fallopian tube disease spectrum (fallopian tube cancer progression)”, including pathology grade, TNM, and clinical stage, containing 30 cases/60 cores.

### 2.2. Clinical Samples Used for Survival Analysis

Formalin-fixed paraffin-embedded (FFPE) tissue samples were collected and sectioned (3 µM) at the Maria Skłodowska-Curie Institute—Oncology Center in Warsaw (Poland). Tissue samples were derived from 108 patients with advanced ovarian cancer who did not receive neoadjuvant chemotherapy. All patients were diagnosed with stage IIIC ovarian cancer (according to Fédération Internationale de Gynécologie et d’Obstétrique; FIGO). Majority of tumors were serous (n = 98) and high-grade (106 grade 3 and grade 4 samples) [26]. Ten samples were classified as undifferentiated and only two samples were grade 2. The mean age of patients was 53.5 ± 10.22 years (range: 29–75 years). The median follow up was 32.85 months (ranging from 4.8 to 177.8 months). Eighty-eight patients died of the disease. All tumor samples were previously evaluated according to TP53 accumulation [27,28]. Complete clinico-pathological characteristics of the group are given in Table 1.

### 2.3. Immunohistochemistry

Tissue arrays and FFPE tissue sections were treated similarly. The only exception was the initial baking applied to tissue arrays for at least 30 min at 60 °C (Heraeus incubator, Kendro laboratory Products, Hanau, Germany) in order to remove excess paraffin. Slides with tissue samples, were de-paraffinized in xylene and rehydrated in decreasing concentrations of ethanol. Antigen retrieval was performed by boiling in 0.01 M citrate buffer (pH 6.0) in microwave (Samsung RE—630 D; 220 V~50 Hz, 1.15 kW). The buffer with slides was brought to a boil four times and allowed to cool for 5 min after each boiling. Subsequently, slides were allowed to cool down in buffer, then were rinsed with PBS three times. Endogenous peroxidase was blocked with 3% hydrogen peroxide, followed by normal horse blocking serum (2.5%; included in ImmPRESS Anti-Rabbit Ig Reagent Kit, MP-7401, Vector Laboratories, Burlingame, CA, USA) for 20 min. Then, sections were incubated with primary antibodies at 4 °C for 12 h. We used rabbit anti-human fibronectin polyclonal antibody (1:3000 dilution, A 0245, Dako, Glostrup, USA) and rabbit anti-human periostin polyclonal antibody (1:200 dilution, ab14041, Abcam, Cambridge, UK). Sections were rinsed with PBS thrice and incubated for 30 min with secondary antibody conjugated to HRP (ImmPRESS Anti-Rabbit Ig Reagent Kit, MP-7401, Vector Laboratories, Burlingame, USA) at room temperature. Immunostaining was performed with 3-3′ diaminobenzidine tetrahydrochloride (DAB), and tissue samples were counterstained with hematoxylin. The sections were examined by light microscopy.

For optimization of IHC procedure we used samples of normal colon tissue and normal lung tissue for anti-fibronectin staining, while for anti-periostin staining optimization, we used normal breast tissue.

### 2.4. Pathological and Immunohistochemical Evaluation of the Samples

The histological assessment of hematoxylin- and eosin-stained tissue sections was performed to evaluate following features: type of the tumor growth (solid, papillary or mixed), angioinvasion (presence of cancer cells within the blood vessels), mitotic activity of cancer cells, inflammatory infiltration, presence of necrosis, calcifications (psammoma bodies), desmoplastic reaction, and anatomical source of the sample. The latter was evaluated based on the presence of peritoneal structures/omental adipose tissue (samples described as P) or ovarian structures (described as O) within the tissue section. Tumor samples without any of these structures were described as T.

After preliminary evaluation of the tissue sections stained with anti-FN1 antibody, we decided to evaluate fibronectin expression only in the stromal compartment of the tumor. Fibronectin expression was scored using three-stage semi-quantitative scale. Score was assigned as follows: 1 (either no staining or single stained connective fibers), 2 (moderate quantity of stained connective fibers) to 3 (strongly stained bunches of connective fibers). The expression of periostin was evaluated separately in cancer cells and in the tumor stroma. A semi-quantitative two-stage scale was used for the assessment of the relative expression of periostin in cancer cells: score weak (less than 50% of stained cancer cells within the section) or strong (more than 50% of stained cancer cells within section). For the assessment of periostin expression in the tumor stroma a three-stage quantitative scale was used: score ranged from 1 (less than 30% of stained connective fibers), 2 (30–60% of stained connective fibers), to 3 (more than 60% of stained connective fibers). All samples were reviewed and scored independently by two researchers, including an experienced pathologist. The slides were scanned into whole slide images using Pannoramic 250 Flash II Scanner.

### 2.5. Statistical Analysis

Statistical analysis was carried out using Statistica version 12 (StatSoft Poland). Overall survival was calculated from the date of diagnosis to the date of death or last follow-up. Disease free survival was calculated for patients with complete response to the first-line chemotherapy, as a time without symptoms. Survival data were plotted as Kaplan–Meier curves, while the log-rank test was used to compare survival between groups. Associations between protein expression and clinic-pathological variables were studied by chi-square test. The independent prognostic significance of protein expression was evaluated using Cox’s proportional hazard model analysis (univariate and multivariate analysis).

## 3. Results

### 3.1. Fibronectin

#### 3.1.1. Expression of Fibronectin in Ovarian Cancer Samples

**Figure 1:**
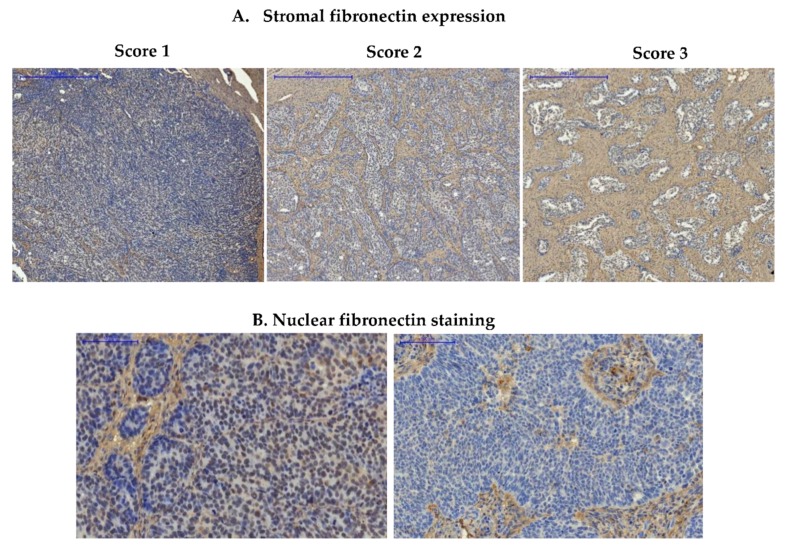
Immunohistochemical detection of fibronectin in ovarian cancer samples. (**A**) Images show representative examples of stromal fibronectin staining, scored as 1 (weak expression), 2 (moderate expression), and 3 (strong expression); (**B**) Images show a representative example of nuclear staining in cancer cells (image on the left) and lack of nuclear staining in cancer cells (image on the right); Pannoramic 250 Flash II Scanner, scale bar: 500 µm (upper panel) and 100 µm (lower panel).

Fibronectin expression was first evaluated in the series of 108 ovarian cancer samples for which we had complete clinico-pathological data, including survival. The majority of these samples were derived from high-grade serous ovarian carcinoma (see Section 2.2). Fibronectin expression was observed mainly in the tumor stroma, where anti-FN1 staining resulted in clear visualization of fibrous structures of the extracellular matrix (ECM) (Figure 1 and Supplementary Figure S1; Supplementary Figure S1 contains paired IHC and H&E images). Twenty-eight samples (25.9%) had strong fibronectin expression (score 3), 64 samples (59.3%) had moderate expression (score 2), while 16 samples (14.8%) showed weak expression (score 1). We also noticed weak nuclear staining of fibronectin in cancer cells. However, this was present in only a few sections, and we decided to exclude this factor from further analyses.

**Figure 2:**
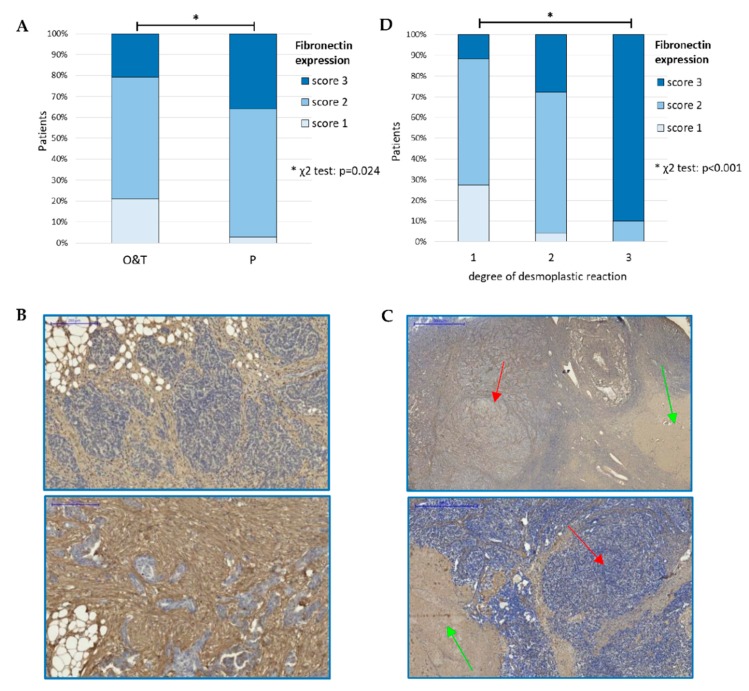
(**A**). Correlation between fibronectin expression and the anatomical source of tumor specimen (χ^2^ test, *p* = 0.024) *p*—samples derived from peritoneal/omental metastatic disease; O—primary tumors (samples containing visible ovarian structures); T—samples containing only tumor tissue; (**B**). Examples of specimens derived from omental metastatic disease (P) with strong fibronectin expression; (**C**). Examples of specimens containing ovarian structures (O), green arrows indicate whitish corpuscles, red arrows indicate nests of cancer cells; (**D**). Correlation between fibronectin expression and the degree of desmoplastic reaction (χ^2^ test, *p* < 0.001). *—denotes *p* < 0.05.

We found significant correlation between fibronectin expression and the source of tumor sample. We compared samples derived from omental metastatic disease (P, samples comprising peritoneal/omental structures; Figure 2B and Supplementary Figure S2B) with primary tumors: samples comprising visible ovarian structures (marked as O) and samples containing only tumor tissue (T). These two types of samples (O and T) showed similar proportion of weak, moderate and strong fibronectin staining, thus they were merged into one group (termed O & T). Among the specimens derived from omental metastatic disease (P) more samples showed moderate and strong fibronectin expression than among remaining (O & T) samples (61% & 36% vs. 58% & 21%; χ^2^ test, *p* = 0.024) (Figure 2A and Supplementary Figure S2A; Supplementary Table S1).

We also found an association between fibronectin expression and the degree of desmoplastic reaction (χ^2^ test, *p* < 0.001) (Figure 2D). Desmoplasia refers to the pathologic expansion of stroma, including overproduction of ECM proteins, increased proliferation of stromal cells and disorganization of ECM structure [29]. We scored desmoplasia using a three-step scale (score 1, 2, and 3). In our series of samples, the majority of tumors with the highest degree of desmoplasia (score 3) had either strong or moderate expression of fibronectin (Supplementary Table S1).

There was no correlation of fibronectin expression with age, size of the residual disease, tumor grade, platinum sensitivity, TP53 accumulation, response to chemotherapy, type of tumor growth (solid versus papillary), mitotic activity, inflammatory infiltration, presence of necrosis, and calcifications in the tumor (Supplementary Table S1).

#### 3.1.2. Prognostic Significance of Fibronectin in Advanced Ovarian Cancer

**Figure 3:**
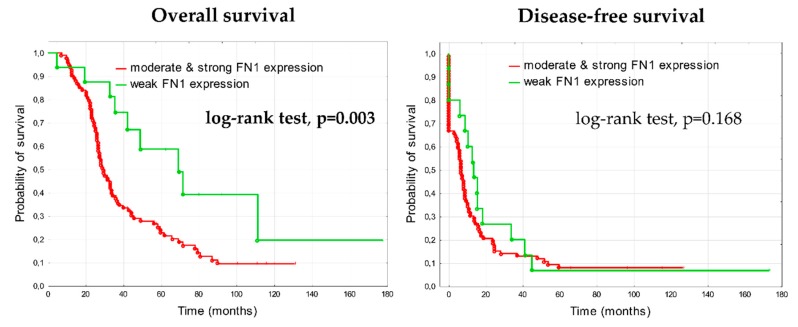
Kaplan-Meier analysis of overall survival and disease-free survival in 108 patients with advanced ovarian cancers stratified by stronger fibronectin expression (score 2 & 3) versus weak fibronectin expression (score 1) in the tumor stroma.

In the analysis of clinical outcome, patients with higher fibronectin expression (score 2 or 3) demonstrated significantly shorter overall survival than patients with weak (score 1) fibronectin expression (log-rank test, *p* = 0.003) (Figure 3). The median overall survival was 67 months in the group with weak expression of fibronectin (4.8–177.8 months, lower quartile 35 months, upper quartile 100 months), whereas in the group with higher expression of fibronectin the median overall survival was 29 months (7.3–131 months, lower quartile 23 months, upper quartile 57.5 months). The difference in relation to DFS was not statistically significant between groups (the median DFS was 13.6 months in group with weak fibronectin expression and 6.8 months in group with higher fibronectin expression).

#### 3.1.3. Expression of Fibronectin on Tissue Arrays

**Figure 4:**
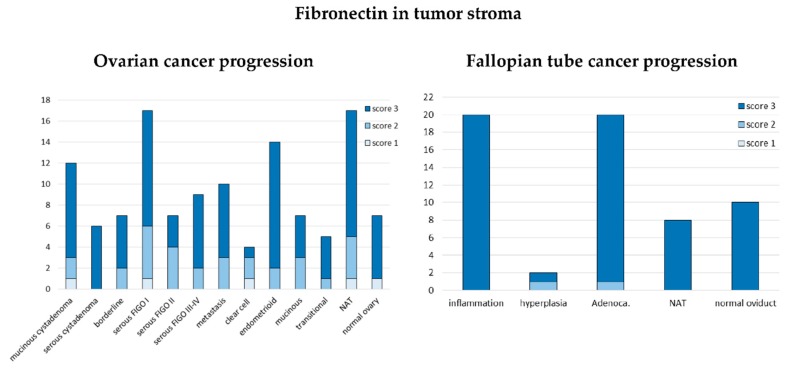
Expression of fibronectin in tissue arrays. The y axis represents the number of samples. NAT—normal tissue adjacent to the tumor.

We also analyzed fibronectin expression using the above described tissue arrays (US Biomax, Inc.). Strong (score 3) stromal fibronectin expression was observed in all samples derived from fallopian tube, except one out of two hyperplasia samples and one out of 20 adenocarcinoma samples; both these outlier samples had moderate (score 2) fibronectin expression (Figure 4). Compared to the fallopian tube, ovarian samples showed wider spectrum of fibronectin expression, however also with prevalence of strong fibronectin staining (score 3). We observed a trend towards more samples with weak and moderate fibronectin staining (score 1 or 2) among low-advanced (FIGO I-II) cancers than among highly advanced ones (FIGO III-IV), however, it was insignificant. Another trend was also visible: the highest number of strongly stained samples (score 3) among endometrioid cancers, while the lowest among clear cell cancer. However, the groups were too small (14 and 4 samples) to draw definite conclusions (Figure 4).

### 3.2. Periostin

#### 3.2.1. Expression of Periostin in Advanced Ovarian Cancers

**Figure 5:**
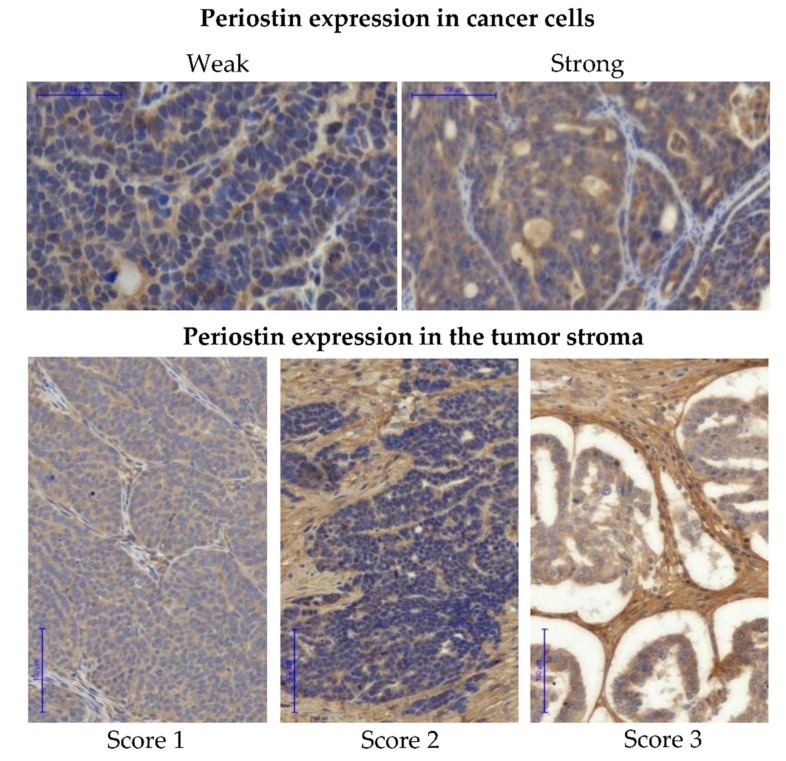
Immunohistochemical detection of periostin. Periostin expression was evaluated separately in cancer cells (upper panel) and in the tumor stroma (lower panel). Images show IHC staining in cancer cells scored either as “weak” or “strong” and IHC staining of stromal periostin scored 1–3. Pannoramic 250 Flash II Scanner, scale bar: 50 µm and 100 µm.

**Figure 6:**
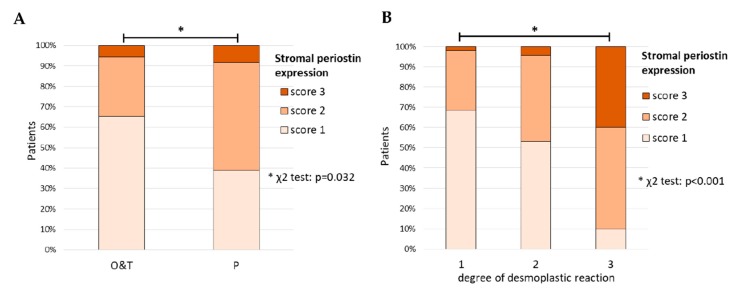
(**A**) Correlation between periostin expression and the anatomical source of tumor specimen (χ^2^ test, *p* = 0.032). P—samples derived from peritoneal/omental metastatic disease; O—primary tumor samples (containing visible ovarian structures); T—samples containing only tumor tissue; (**B**) Correlation between stromal periostin expression and the degree of desmoplastic reaction (χ^2^ test, *p* < 0.001). *—denotes *p* < 0.05.

The protein expression of *POSTN* was first evaluated in the series of 108 ovarian cancer samples (mainly HGSOC) for which we had complete clinico-pathological data, including survival (see chapter 2.2). Periostin could be detected both in cancer cells and in the tumor stroma. Periostin expression in each location was analyzed separately (see Materials and Methods) (Figure 5 and Supplementary Figure S3). Similar to the case of fibronectin, the expression of periostin in tumor stroma was found to be associated with the source of tumor samples (Figure 6A).

We observed more tumors with stronger periostin expression (score 2 or 3) among the samples derived from omental metastatic disease (P) than in remaining samples (53% & 8% vs. 29% & 5%; χ^2^ test, *p* = 0.032). The latter group consisted of tumor sections either containing visible structures of the ovary (O) or without such structures (T) (Supplementary Table S2). Higher expression (score 2 or 3) of periostin in the tumor stroma, as well as in cancer cells, was associated with higher degree of desmoplastic reaction (χ^2^ test p < 0.001 and *p* = 0.037, respectively) (Figure 6B, Supplementary Table S2).

Additionally, there was an association between periostin expression in cancer cells and the presence of calcifications. Majority of samples containing calcifications had strong periostin expression in cancer cells (χ^2^ test, *p* = 0.019). There was no difference in relation to age, size of the residual disease, tumor grade, platinum sensitivity, TP53 accumulation, chemotherapy response, growth type, mitotic activity, inflammatory cells infiltration, and necrosis (Supplementary Table S2).

#### 3.2.2. Prognostic Significance of Periostin in Advanced Ovarian Cancer

**Figure 7:**
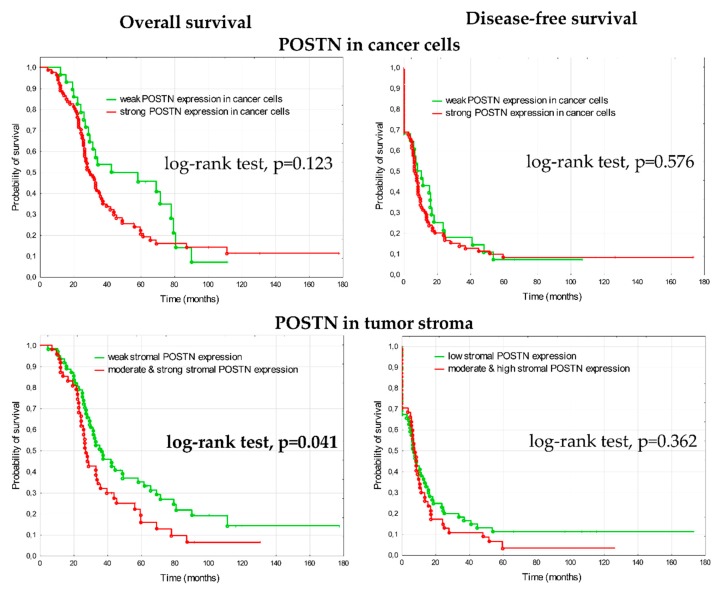
Kaplan–Meier analysis of overall survival (OS) and disease-free survival (DFS) in 108 patients with advanced ovarian cancer stratified by weak versus strong periostin expression in cancer cells and weak (score 1) versus higher (score 2 + 3) periostin expression in the tumor stroma.

In the analysis of clinical outcome, patients with higher periostin expression in the tumor stroma (score 2&3, n = 47) had shorter overall survival than patients with weak expression (score 1, n = 61) (Figure 7). The median OS was 36 months in the group with weak expression of periostin (4.8–177.8 months, lower quartile 25.5 months, upper quartile 77.5 months), whereas in the group with higher expression of periostin the median overall survival was 27 months (7.3–131.2 months, lower quartile 22.5 months, upper quartile 45.6 months). The difference in relation to DFS was not statistically significant between groups; the median DFS was 7.2 months in group with weak periostin expression and 7.7 months in group with higher periostin expression. There were no statistically significant differences between groups with weak and strong periostin expression in cancer cells, in relation to OS and DFS.

#### 3.2.3. Expression of Periostin in Tissue Arrays

**Figure 8:**
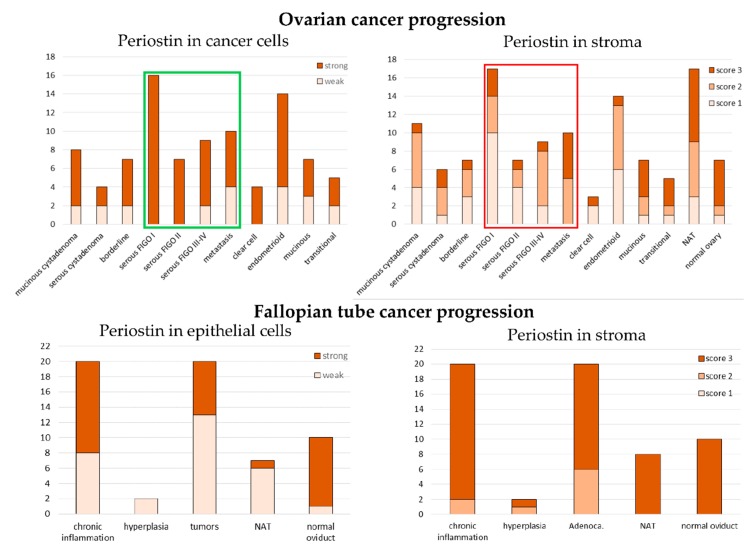
Expression of periostin in tissue arrays. The y axis represents the number of samples. NAT—normal tissue adjacent to the tumor.

Tissue arrays (US Biomax, Inc.) were used to asses periostin expression in different stages of progression of ovarian and fallopian tube epithelial tumors and different histological types of ovarian cancer (Figure 8). When we looked at periostin expression in cancer cells, we observed that all early stage serous ovarian cancers (FIGO I-II) showed strong staining, while some percentage of FIGO III-IV and metastatic samples showed weak staining. The difference between these groups was statistically significant (χ^2^ test *p* = 0.004). An inverse correlation was observed for the stromal expression of periostin in serous cancers. More samples with stronger stromal expression (score 2 or 3) were among FIGO III-IV and metastatic samples than among FIGO I-II samples. This difference was statistically significant (χ^2^ test *p* = 0.005). Samples derived from fallopian tube, both normal and diseased, demonstrated higher expression of periostin in the stromal than in the epithelial compartment.

### 3.3. Prognostic Significance of Clinico-Pathological Features

**Figure 9:**
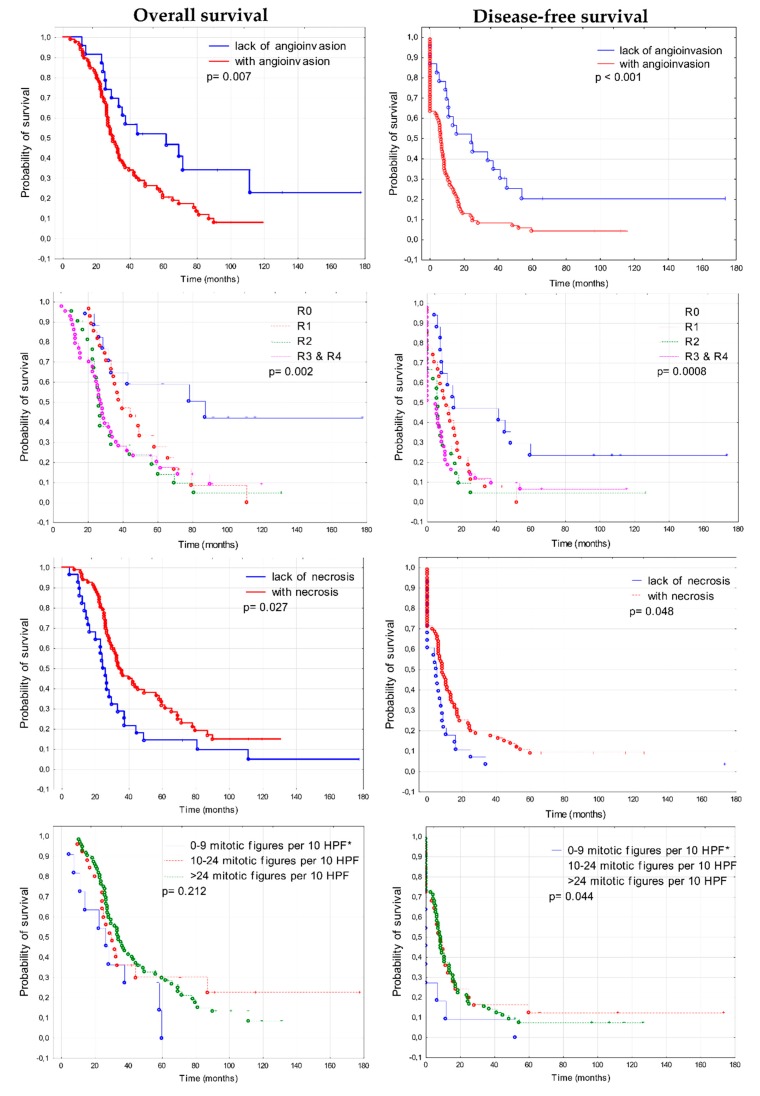
Kaplan–Meier analysis of overall survival (OS) and disease-free survival (DFS) in 108 ovarian cancer patients in relation to clinico-pathological features. * HPF—high power field; R—residual disease: R0 = 0 cm, R1 < 0.5 cm, 0.5 cm ≤ R2 < 2 cm, 2 cm ≤ R3 ≤ 5 cm, R4 > 5 cm.

The Kaplan–Meier analysis performed in the series of 108 ovarian cancer samples showed that several clinico-pathological features had prognostic significance. Such features included: size of residual disease, angioinvasion (presence of cancer cells in blood vessels), necrosis and mitotic activity (Figure 9). Patients who were classified as R0 group (R0—no residual tumor), had significantly longer survival in comparison to patients with residual tumor of any size (χ^2^ test, *p* = 0.002 for OS, *p* = 0.0008 for DFS). Median OS for patients without residual disease was 78.65, while for patients with residual disease of any size, it was 29.76. Median DFS was 15.16 and 6.35 months, respectively. These observations are concordant with the results of large multivariate analyses that indicate improved progression-free and overall survival for group of patients with complete resection, compared to groups with the so-called optimal (between 0.1 and 1 cm) and suboptimal cytoreduction (*p* < 0.0001) [30]. Since 2017, European Society of Gynecological Oncology (ESGO) guidelines recommend that the aim of the frontline surgery should be to achieve the complete resection of macroscopic residuals of the disease (complete cytoreduction) [31].

Subsequently, we found that patients who had tumors with angioinvasion demonstrated shorter survival in comparison to patients whose tumors showed no signs of angioinvasion (log-rank test, *p* = 0.007 for OS, *p* < 0.001 for DFS). Median OS was 50.53 for patients without angioinvasion and 29.76 for patients with angioinvasion in their tumors. Median DFS was 19.98 months and 6.35 months, respectively. Interestingly, patients with necrotic tumors had longer survival than patients with tumors without necrosis (log-rank test, *p* = 0.027 for OS, *p* = 0.048 for DFS). Median OS was 34.37 and 24.5 month, respectively, while median DFS was 8.5 months, respectively [32]. The mitotic activity of cancer cells correlated only with DFS. Patients with increased mitotic activity had longer DFS than patients with lower mitotic activity (χ^2^ test, *p* = 0.044). Other factors such as age, the accumulation of TP53, type of tumor growth, inflammatory infiltrate, presence of calcifications, and degree of desmoplasia showed no correlation with survival (Supplementary Figure S4).

### 3.4. Prognostic Significance of Fibronectin 1 and Periostin (FN1&POSTN Score)

Next, we examined whether fibronectin and periostin expression in tumor stroma, analyzed jointly, showed an association with overall survival. We used a combined score (FN1&POSTN score) calculated by adding together individual scores for fibronectin and periostin expression (see Table 2).

**Figure 10:**
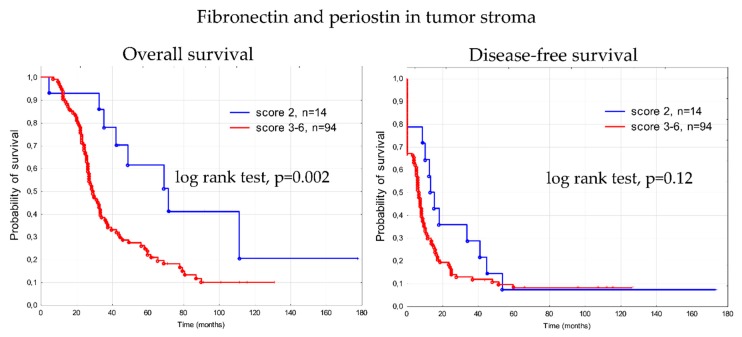
Kaplan-Meier analysis of overall survival (OS) and disease-free survival (DFS) in 108 patients with advanced ovarian cancer stratified by combined FN1&POSTN scores (score 2 versus score 3–6).

Kaplan–Meier curves of overall survival and disease-free survival for 108 patients, stratified by combined scores for FN1&POSTN expression, are shown in Supplementary Figure S5. Median OS and OS range in all five groups according to FN1&POSTN score are given in Table 2. Patients with the lowest score (score 2) demonstrated longer OS in comparison to all remaining patients (median OS for score 2 was 55.88 months, for score 3–6—it was 29.37 months; Figure 10). The difference was statistically significant (log rank test, *p* = 0.002). However, these two groups of patients were not uniform. Unexpectedly, one patient with a score 2 (indicating favorable prognosis) tumor had the shortest OS (4.8 months) of the entire cohort of 108 patients. Likewise, one patient with score 6 (indicating the worst prognosis) had the second longest OS (131.17 month) in the whole cohort. These exceptions indicate that the FN1&POSTN score behaves similarly to classical prognostic factors. It is also observed that some patients having good prognosis based on clinical criteria, progress quickly and die early, while some patients with bad prognosis live unexpectedly long.

All factors revealed in Kaplan–Meier analysis as significantly associated with survival were further assessed using the univariate and multivariate Cox’s proportional hazards analysis (Table 3 and Table 4). The joint score of FN1&POSTN expression appeared to be an independent prognostic factor in terms of OS, as well as residual tumor size, presence of angioinvasion, and necrosis. According to DFS, only residual disease, angioinvasion, and necrosis were confirmed as independent prognostic factors.

## 4. Discussion

The results of our study add to some previous observations that fibronectin and periostin may have prognostic significance in ovarian cancer. The exact mechanism of this phenomenon remains to be elucidated. However, some explanation comes from the fact that both of these proteins are involved in shaping the structure and regulating function of the extracellular matrix (ECM). ECM is a major component of the tumor microenvironment, together with the vascular network, fibroblasts, immune and stem cells, etc. This complex environment is deeply involved in the control of tumor progression.

### 4.1. The Role of Fibronectin in Ovarian Cancer

Cellular fibronectin has broad biological activity, serving as a structural scaffold and playing an important role in the regulation of cell adhesion and motility. The main fibronectin-specific integrin, which is present on most cells, consists of two subunits: α5 and β1 (reviewed in: [33]). Importantly, fibronectin–integrin interactions proved not only able to supply the mechanical force necessary for cell migration, but also to be engaged in signal transduction. Thus, although the role of fibronectin in cancer is only partially understood, there are multiple potential mechanisms emerging, through which this role may be exerted. It seems that the main mechanism is based on the activation of the focal adhesion kinase (FAK). Downstream FAK/Src signaling can have very diverse cellular effects: from the induction of epithelial to mesenchymal transition (EMT) and acquisition of invasive phenotype, to providing proliferative signaling and even inducing antiapoptotic activity (reviewed in: [6,9]). It was confirmed by Mitra et al. that such signaling occurs in ovarian cancer cells: fibronectin binding to α5β1-integrin leads to a direct association of α5-integrin with the c-Met kinase, activating it in a ligand-independent manner. Subsequently, c-Met associates with Src, and activates Src and FAK. This mechanism may promote the invasion and metastasis of ovarian cancer cells [34].

Moreover, fibronectin seems to take part in the cross-talk between ovarian cancer cells and other cells of the tumor microenvironment. In our work, we observed that tumor samples derived from omental metastatic disease demonstrated significantly stronger expression of fibronectin than samples of primary tumors. Similar results are returned when querying *FN1* using the CSIOVDB transcriptomic microarray database which integrates gene expression data from 48 published datasets (comprising 3,431 epithelial ovarian cancer samples) [35]: it could be found that *FN1* mRNA is significantly increased in metastatic peritoneal tumors compared with primary ovarian tumors (*p* = 2.22 × 10^−6^ for comparison between classes: “Tumor” vs. “Peritoneal”). Further, Kenny et al. showed that fibronectin expression was higher in the omental metastases than in the primary tumors [36]. These authors performed a series of very elegant in vitro and in vivo experiments, showing that TGF-β produced by ovarian cancer cells can activate a TGF-β receptor/RAC1/SMAD-dependent signaling pathway in the mesothelial cells, what induces EMT and results in overexpression of fibronectin. Fibronectin, in turn, promotes the adhesion and invasion of cancer cells. This mechanism may be responsible for the preferential implantation of ovarian cancer cells on the surface of omentum, through interactions with mesothelial cells lining its surface. Such a hypothesis is supported by the observation that blocking fibronectin production in primary mesothelial cells in vitro decreases the adhesion, invasion, and proliferation of ovarian cancer cells. A pro-tumourigenic role for fibronectin is also confirmed by the fact that SKOV3 ovarian cancer cells have reduced invasiveness and metastatic potential in fibronectin knockout mice [36].

Another key component of the cancer microenvironment are cancer associated fibroblasts (CAFs). Yeung et al. examined the gene expression profile of microdissected ovarian cancer cells and CAFs derived from HGSOC tumors [37]. They observed that cancer cells overexpress TGF-β, while CAFs can respond through activated Smad signaling. Thus, it can be speculated that similar relationship to those described between ovarian cancer and mesothelial cells, also pertain to CAFs.

Increased fibronectin expression is generally regarded as a marker of mesenchymal phenotype. Gene expression data from CSIOVDB indicate that higher *FN1* mRNA significantly correlates with an EMT score. Accordingly, the highest *FN1* expression is observed in the mesenchymal subtype of ovarian cancer which is characterized by the worst prognosis [38].

In this work, we have demonstrated that stronger stromal fibronectin expression in the tumor is associated with the shorter OS of patients with advanced ovarian cancer. Prognostic significance of fibronectin was first noticed by Franke et al. [22] who analyzed 211 tumors of different histological type to observe that higher fibronectin expression correlated significantly with worse OS and was an independent prognostic factor (*p* = 0.009). Also in the CSIOVDB, *FN1* mRNA levels higher than the median are significantly associated with both, reduced OS (*p* < 0.0001) and reduced DFS (*p* < 0.0001). In the multivariate analysis *FN1* expression retains as an independent predictor of DFS.

CSIOVDB also indicates that higher *FN1* expression is related with resistance to the first line chemotherapy. It was also observed by others that the level of fibronectin (as well as matrix metalloproteinase 9) was significantly higher in tumor samples and in the ascites fluid of the recurrent ovarian cancer patients and in the group of patients who died from the disease, as compared to the non-recurrent cases [23]. These data further support the connection between fibronectin expression and worse prognosis in ovarian cancer.

CSIOVDB provides also the information that higher *FN1* expression is related to serous histology, with higher FIGO stage, and higher grade. These results were not confirmed in our tissue microarray analysis (see Figure 4). Discrepancies between CSIOVDB and our results may be partially due to the fact that we analyzed protein expression, while CSIOVDB supplies the data on mRNA quantity; these are two distinct levels of gene expression, not always coherent.

There have also been attempts to use fibronectin for diagnosis and treatment. It was shown that a nine-biomarker diagnostic panel, including fibronectin, could better discriminate ovarian cancers from benign ovarian masses than the OVA1 test (at a threshold sensitivity of 90%, the nine-marker panel gave 88.9% specificity, compared to 63.4% for the OVA1) [39].

Making use of fibronectin in therapy is based on the assumption that, in adults, alternatively-spliced fibronectin variants containing extra domains A and B (EDA/EDB) are primarily limited to sites of malignancy [9,40]. One strategy which has been tested, utilizes recombinant human antibody L19 (specific to the EDB) fused with cytokines such as interleukin 2 (IL2) or tumor necrosis factor α (TNFα). As EDB is strongly expressed in stromal and neo-vascular structures during cancer progression, this strategy could deliver therapeutic agents directly to the tumor and reduce side effects of the systemic activity of these cytokines [41]. A phase I/II clinical trial with L19-IL2 in patients with solid tumors allowed to evaluate safety, tolerability, recommended phase II dose and showed early signs of activity [42]. However, another phase I/II clinical trial with L19-TNF in patients with solid tumors did not showed objective tumor responses. Transient stable disease occurred in 19 of 31 evaluable patients [43].

### 4.2. Periostin Significance in Ovarian Cancer

Periostin, also called osteoblast-specific factor 2 (OSF-2), is a multifunctional ECM glycoprotein. Periostin is capable of binding to multiple integrin receptors (αvβ3, αvβ5, α6β4), thus affecting the regulation of the intracellular signaling pathways associated with protein kinases PI3K/AKT and FAK. This protein plays a role in the adhesion and migration as well as in EMT and remodeling of the extracellular matrix. Periostin is also implicated in the metastases of cancer cells and lymph- and angiogenesis (reviewed in: [16,44,45]. Some data indicate that periostin may exert an important role in ovarian cancer and may be potentially useful as a prognostic and predictive biomarker.

In our previous microarray study, we found the 96-gene prognostic signature including *POSTN* which had the highest change in mRNA level (fold change 21.69) between two groups of patients with significantly different OS [5]. Further, the output from CSIOVDB indicates that *POSTN* mRNA level is significantly associated with OS and DFS. However, in the multivariate analysis periostin is an independent prognostic factor only with regard to DFS. Further, Karlan et al. (2014) identified a prognostic gene signature containing *POSTN* [24]. They found that genes from this signature were related with TGFβ- and integrin-signaling. Patients with an upregulated *POSTN/TGFβ* signature had significantly shorter OS than patients with lower expression of these genes.

Another microarray study performed by Ryner et al. (2015) revealed that POSTN was overexpressed in primary resistant ovarian cancers, while downregulated in chemosensitive ones [46]. In their study, *POSTN* was found to be regulated coordinately with *FAP, LOX, TIMP3* and *COL4A1*, similarly like in our previous study [5]. We also share a common observation that a higher degree of desmoplasia is correlated with the higher stromal expression of periostin. In addition, Ryner et al. observed that periostin is more highly expressed in recurrent tumors than in non-recurrent ones. Using in situ hybridization and IHC, they revealed that periostin was predominantly produced by CAFs.

Sung et al. (2015) evaluated periostin expression by IHC, using a tissue microarray with 308 samples derived from ovarian tumors of different histology and stages [25]. They observed a correlation of stromal periostin expression with more advanced FIGO stage, suboptimal cytoreduction, tumor recurrence, and survival: patients with high periostin expression in tumor stroma had shorter OS and DFS. Similarly, as in our study, there was no correlation between periostin expression in cancer cells and survival.

### 4.3. FN1&POSTN Score

Both analyzed proteins, fibronectin and periostin, are engaged in similar cellular processes and are indicated as related to the survival of ovarian cancer patients. In addition, we have shown that both proteins have higher expression in omental metastases than in primary tumors, which supports their role in metastatic process. There are not many data concerning their mutual expression, however it was postulated that periostin can be a modulator of fibronectin production during gingival healing [47]. Thus, it is possible that both proteins can be expressed coordinately or within short time interval. For these reasons, we decided to check whether the combined score based on their immunohistochemical staining in the tumor stroma (FN1&POSTN score) will perform better than individual scores for each protein. Indeed, in the multivariate analysis, this score was significant (*p* = 0.044), with a *p*-value slightly worse than that for fibronectin only (*p* = 0.037), but better than periostin only, which was insignificant. When analyzing all five survival curves for patients with scores of 2, 3, 4, 5, and 6 separately, it could be noticed that this result was spoiled by the occurrence of two patients who behaved unexpectedly. One patient with a score 2 tumor (indicating favorable prognosis) had the shortest OS (4.8 months) out of the entire cohort of 108 patients. Likewise, one patient with score 6 (indicating worst prognosis) had the second longest OS (131.17 month) out of the whole cohort. These exceptions cause the FN1&POSTN score to behave similarly to classical prognostic factors, for which it is also frequently observed that some patients with good prognosis progress quickly and die early, while some patients with bad prognosis have unexpectedly long OS. This indicates that further studies are necessary to find molecular markers (or their panels) which will enable more precise prognosis.

### 4.4. Other Prognostic Factors

Angioinvasion is widely recognized as an independent negative prognostic factor related to survival, and this was confirmed in our analysis. More surprising was our finding that the presence of necrosis in the tumor could be a positive prognostic factor. There are some indications that necrosis occurring after chemotherapy is indicative of tumor response and has positive prognostic value [32], however, our patients were not subject to neoadjuvant chemotherapy.

One of the most important prognostic factors in ovarian cancer is the size of the residual tumor (R) left after primary surgery. However, there has been a long-lasting discussion about the threshold corresponding to the optimal surgical debulking status, i.e., one having a significant influence on the OS. Our results showed that patients without any residual disease (R0) live significantly longer than all remaining patients (R1–R5). These observations are in accordance with the current knowledge and recommendations of the European Society of Gynaecologial Oncology, where complete tumor resection at upfront debulking is the main goal of surgery. It is also recommended to select patients in whom complete tumor resection is feasible. Otherwise, neoadjuvant treatment and interval debulking surgery is recommended [48].

## 5. Conclusions

Our results indicate that fibronectin and periostin have a prognostic significance in ovarian cancer. The expression of fibronectin and periostin was also associated with the source of ovarian tumor sample: metastases showed higher expression of these proteins than primary tumor samples. The elevated expression of these proteins was also more common in fallopian tube cancers than in ovarian cancers.

Overall, our results support the role of the cancer microenvironment in tumor progression and prognosis. Our results also indicate, together with the results obtained by others [9], that fibronectin and periostin play the role of extracellular drivers of malignancy.

## Figures and Tables

**Table 1:** Characteristics of patients and tumor samples.

Characteristics	(Total)	Number of Samples							
Residual tumor ^1^	(108)	R0	17	R1	27	R2	21	R3	43
CHT response (acc. to RECIST) ^2^	(108)	CR	74	PR	31	NC	1	P	2
Histopathological type	(108)	serous	97	undifferentiated	10	other	1		
Tumor grade	(108)	G2	2	G3	76	G4 *	30		
Platinum sensitivity ^3^	(108)	Highly sensitive	22	Moderately sensitive	42	Resistant	44		
TP53 accumulation	(108)	Yes	68	No	40				
Age	(108)	≤54 years	56	>54 years	52				

^1^ Residual tumor size: R0 = 0 cm, R1 < 0.5 cm, R2—between 0.5 cm and 2 cm, R2 ≥ 2 cm; ^2^ Chemotherapy (CHT) response, described as clinical status of the patient after first-line treatment: CR—complete response, PR—partial response, NC—no change, P—progression; RECIST—Response Evaluation Criteria in Solid Tumors; ^3^ Tumors were classified as highly sensitive when disease free survival (DFS) > 732 days, moderately sensitive when 732 days > DFS > 180 days, and resistant when DFS < 180 days *—classification criteria given by Barber [26].

**Table 2:** Fibronectin and periostin analyzed altogether.

Stromal FN1	StroMal POSTN	FN1&POSTN Score	No. of Samples	Median OS Months
1	1	2	14	55.88 (range 4.8–77.83)
1	2	3	2	30.92 (range 10.7–119.93)
2	1		42	
2	2	4	21	28.77 (range 7.3–100.83)
1	3		0	
3	1		5	
2	3	5	1	26.55 (range 11.53–91.53)
3	2		17	
3	3	6	6	42.23 (range 9.87–131.17)

**Table 3:** Univariate and multivariate analysis of overall survival.

Feature	Univariate Analysis		Multivariate Analysis	
	HR (95% CI)	*p* Value	HR (95% CI)	*p* Value
FN1 & POSTN				
Score 3–6 vs. Score 2	2.54 (1.22–5.28)	0.012	2.16 (1.02–4.60)	0.044
Angioinvasion				
Present vs. Absent	1.98 (1.13–3.47)	0.017	1.76 (0.99–3.15)	0.054
Necrosis				
Present vs. Absent	0.57 (0.36–0.89)	0.016	0.51 (0.32–0.82)	0.006
Residual Disease				
Present vs. Absent	2.87 (1.43–5.76)	0.003	2.39 (1.18–4.83)	0.015

**Table 4:** Univariate and multivariate analysis of disease-free survival.

Feature	Univariate Analysis		Multivariate Analysis	
	HR (95% CI)	*p* Value	HR (95% CI)	*p* Value
FN1&POSTN				
Score 2 vs. Score 3–6	1.46 (0.81–2.63)	0.203	1.02 (0.55–1.90)	0.93
Angioinvasion				
Present vs. Absent	2.27 (1.35–3.83)	0.002	2.48 (1.42–4.32)	0.001
Necrosis				
Present vs. Lack	0.62 (0.39–0.97)	0.038	0.55 (0.34–0.88)	0.013
Residual Disease				
Present vs. Lack	2.34 (1.29–4.25)	0.005	2.17 (1.18–3.99)	0.012

## Supplementary Materials

The following are available online at https://www.mdpi.com/2073-4409/9/1/149/s1, Figure S1: Immunohistochemical detection of fibronectin in ovarian cancer samples (paired IHC and H&E images), Figure S2: A. Examples of specimens derived from omental metastatic disease with strong fibronectin expression (paired IHC and H&E images); B. Examples of specimens containing ovarian structures (paired IHC and H&E images), Figure S3: Immunohistochemical detection of periostin in cancer cells and in tumor stroma (paired IHC and H&E images), Figure S4: Kaplan-Meier analysis of overall survival (OS) and disease-free survival (DFS) in 108 patients in regard to following clinico-pathological features: age, grade, TP53 accumulation, degree of desmoplastic reaction, type of tumor growth (papillary versus solid versus mixed), inflammatory infiltration, calcifications in the tumor (all nonsignificant), Figure S5: Kaplan-Meier analysis of overall survival (OS) and disease-free survival (DFS) in 108 patients stratified by combined FN1&POSTN score, Table S1: Association between fibronectin expression and clinico-pathological features (χ2 test), Table S2: Association between periostin expression and clinico-pathological features (χ2 test).
