# Supplementary material for: Fibronectin and Periostin as Prognostic Markers in Ovarian Cancer
Source: Cells. 2020 Jan 8;9(1):149. doi: 10.3390/cells9010149 (PMC7016975; doi:10.3390/cells9010149)
Supplement: Supplementary file 1 [file cells-09-00149-s001.pdf]

Supplementary Materials

Fibronectin and periostin as prognostic markers in ovarian cancer

Table S1. Association between fibronectin expression and clinico-pathological features ($\chi 2$ test).

Characteristics	Number of patients		FN1 expression						p-value
			weak (n=16, 14.8%)		moderate (n=64, 59.3%)		strong (n=28, 25.9%)		
	n	%	n	%	n	%	n	%	
**Age**									
**≤ 54 years**	56	51.85	10	17.86	34	60.71	12	21.43	0.433
**> 54 years**	52	48.15	6	11.54	30	57.69	16	30.77	
**Residual disease**									
**No**	17	15.74	4	23.53	8	47.06	5	29.41	0.441
**Yes**	91	84.26	12	13.19	56	61.54	23	25.27	
**Grade***									
**G3**	76	71.70	10	13.16	45	59.21	21	27.63	0.559
**G4**	30	28.30	6	20.00	18	60.00	6	20.00	
**Platinum sensitivity**									
**Highly sensitive (DFS > 24 mths)**	22	20.37	5	22.73	10	45.45	7	31.82	0.285
**Moderately sensitive (24 mths> DFS > 6 mths)**	42	38.89	8	19.05	25	59.52	9	21.43	
**Resistant (DFS < 6 mths)**	44	40.74	3	6.82	29	65.91	12	27.27	
**TP53 Accumulation**									
**-**	40	37.04	7	17.50	23	57.50	10	25.00	0.834
**+**	68	62.96	9	13.24	41	60.29	18	26.47	
CHT response									
**CR**	74	68.52	13	17.57	43	58.11	18	24.32	0.415
**PR**	31	28.70	2	6.45	19	61.29	10	32.26	
**NC or P**	3	2.78	1	33.33	2	66.67	0	0.00	
**Growth type**									
**Papillary**	17	15.74	0	0.00	10	58.82	7	41.18	0.308
**Solid**	34	31.48	6	17.65	20	58.82	8	23.53	
**Mixed**	57	52.78	10	17.54	34	59.65	13	22.81	
**Mitotic activity**									
**1 (0-9/10HPF)**	11	10.19	1	9.09	6	54.55	4	36.36	0.542
**2 (10-24/10HPF)**	25	23.15	3	12.00	13	52.00	9	36.00	
**3 (> 24/10HPF)**	72	66.67	12	16.67	45	62.50	15	20.83	

Characteristics	Number of patients		FN1 expression						p-value
			weak (n=16, 14.8%)		moderate (n=64, 59.3%)		strong (n=28, 25.9%)		
	n	%	n	%	n	%	n	%	
**Source of tumor sample**									
**Ovary (O)**	39	36.11	8	20.51	23	58.97	8	20.51	0.112
**Tumor (T)**	33	30.56	7	21.21	19	57.58	7	21.21	
**Peritoneum (P)**	36	33.33	1	2.78	22	61.11	13	36.11	
**O + T**	72	66.67	15	20.83	42	58.33	15	20.83	**0.024**
**P**	36	33.33	1	2.78	22	61.11	13	36.11	
**Inflammatory infiltration**									
**Weak**	81	75.00	14	17.28	44	54.32	23	28.40	0.18
**Moderately + Strong**	27	25.00	2	7.41	20	74.07	5	18.52	
**Degree of desmoplastic reaction**									
**1**	51	47.22	14	27.45	31	60.78	6	11.76	**< 0.001**
**2**	47	43.52	2	4.26	32	68.09	13	27.66	
**3**	10	9.26	0	0.00	1	10.00	9	90.00	
**Necrosis**									
**-**	28	25.93	4	14.29	17	60.71	7	25.00	0.984
**+**	80	74.07	12	15.00	47	58.75	21	26.25	
**Calcification**									
**-**	78	72.22	12	15.38	50	64.10	16	20.51	0.114
**+**	30	27.78	4	13.33	14	46.67	12	40.00	

*** G2 ignored, because of only two patients with G2 tumors;**
HPF – high power field (400-fold magnification);
CHT response: CR – complete response, PR – partial response, NC – no change, P - progression
O – tumor section containing ovarian structure(s), T – section with tumor tissue only, P – section containing peritoneal/omental structures;
Fibronectin expression: weak – score 1, moderate – score 2, strong – score 3

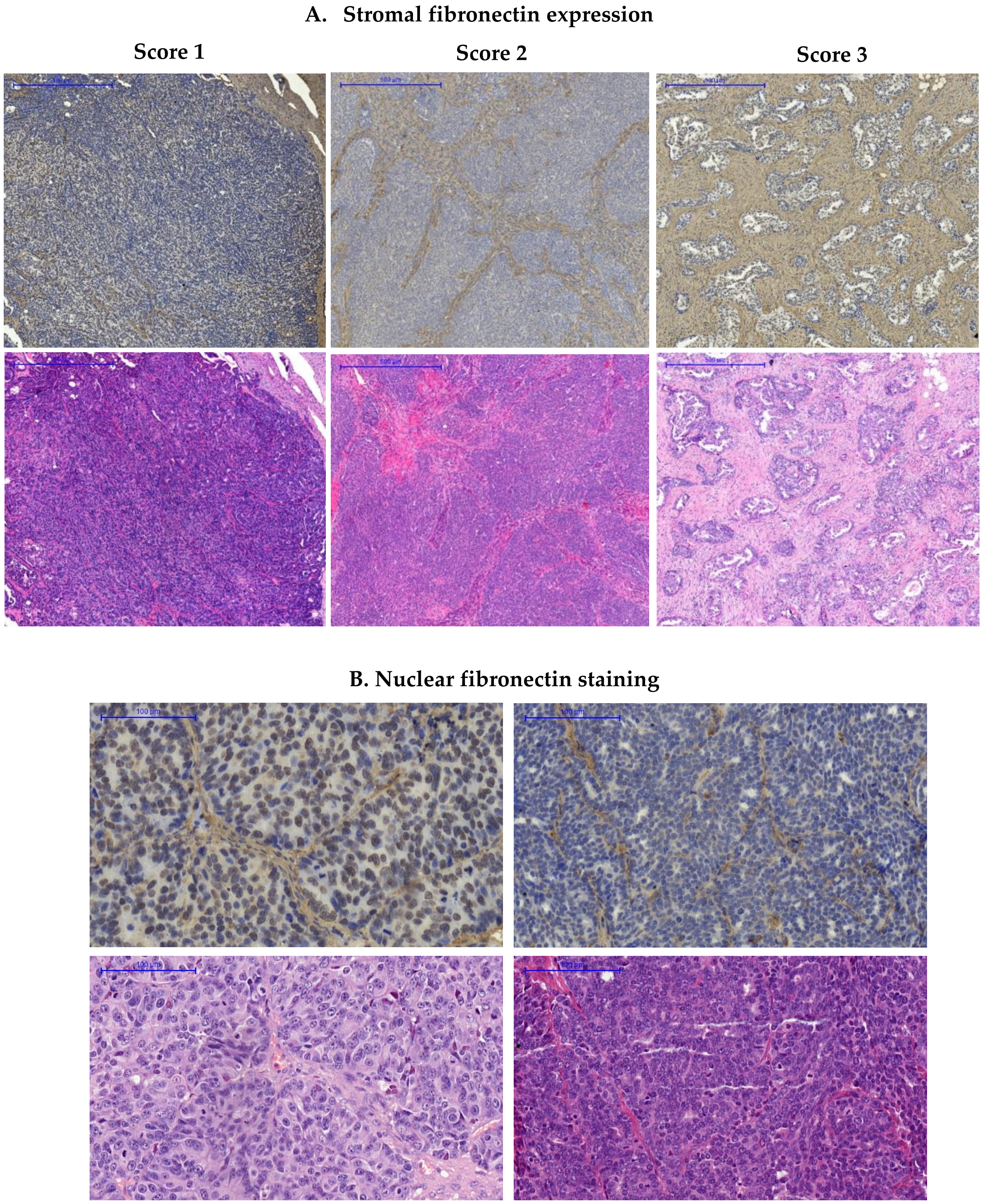

Figure S1. Immunohistochemical detection of fibronectin in ovarian cancer samples. A. IHC images (upper panel) show representative examples of stromal fibronectin staining, scored as 1 (weak expression), 2 (moderate expression), and 3 (strong expression); lower panel contains corresponding H&E images; B. Images in the upper panel show representative example of nuclear anti-FN1 staining in cancer cells (image on the left) and lack of nuclear staining in cancer cells (image on the right). Lower panel contains corresponding H&E images; Pannoramic 250 Flash II Scanner, scale bar: 500 µm (images in A) and 100 µm (images in B).

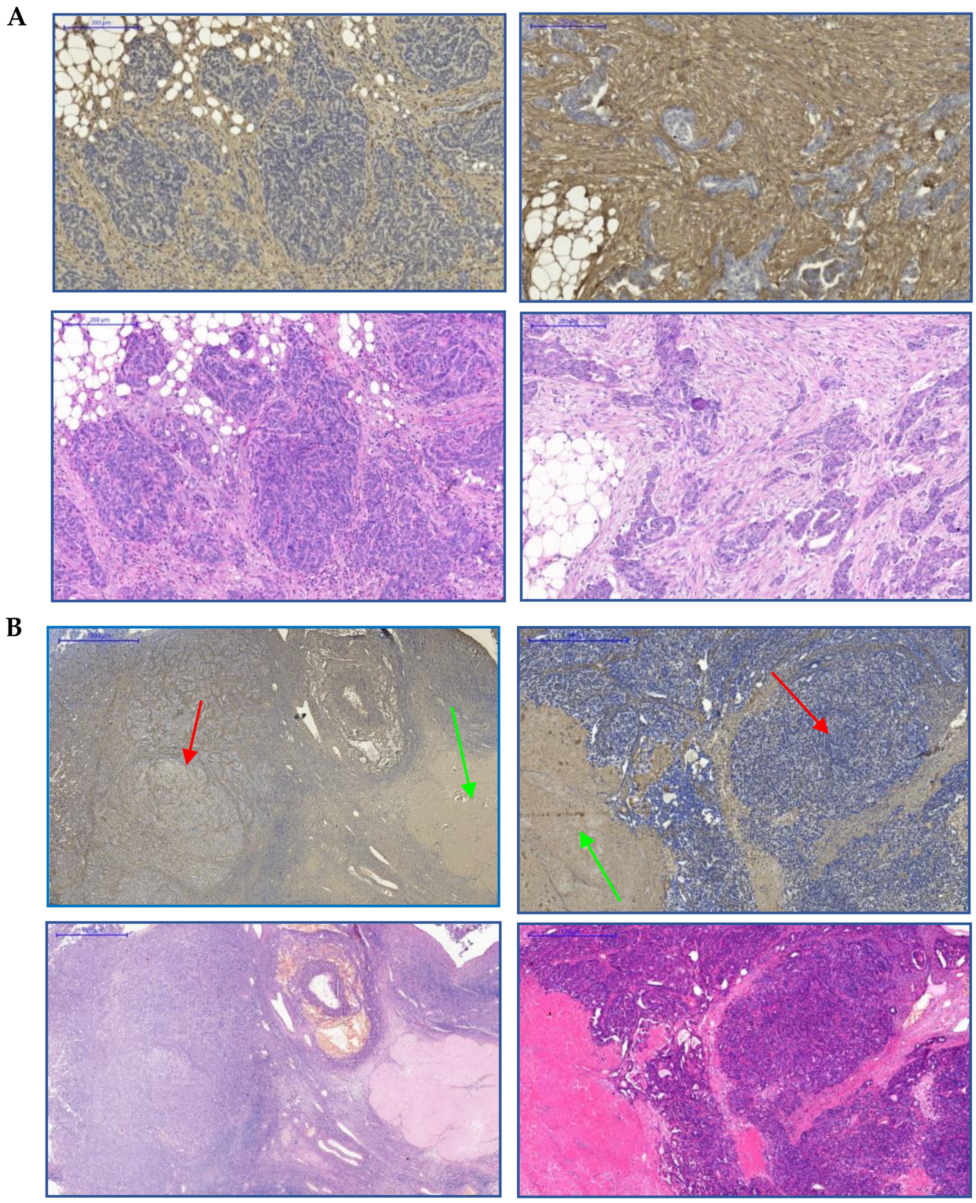

Figure S2. A. Examples of specimens derived from omental metastatic disease (P) with strong fibronectin expression (upper panel) and corresponding H&E images (lower panel); B. Upper panel: examples of specimens containing ovarian structures (O), green arrows indicate whitish corpuscles, red arrows indicate nests of cancer cells; lower panel: correspondinh H&E images. Pannoramic 250 Flash II Scanner, scale bar: 200 µm (images in A), 1000 µm and 500 µm (images in B).

TableS2. Association between periostin expression and clinico-pathological features (χ2 test).

Characteristics	Number of patients		POSTN expression in cancer cells				P value	POSTN expression in tumor stroma						P value
			weak (n=28, 25.9%)		strong (n=80, 74%)			weak (n=61, 56.5%)		moderate (n=40, 37%)		strong (n=7, 6.5%)		
	n	%	n	%	n	%		n	%	n	%	n	%	
**Age**														
≤ 54 years	56	51.85	15	26.79	41	73.21	0.832	33	58.93	20	35.71	3	5.36	0.817
> 54 years	52	48.15	13	25.00	39	75.00		28	53.85	20	38.46	4	7.69	
**Residualdisease**														
No	17	15.74	7	41.18	10	58.82	0.118	13	76.47	3	17.65	1	5.88	0.175
Yes	91	84.26	21	23.08	70	76.92		48	52.75	37	40.66	6	6.59	
**Grade***														
G3	76	71.70	19	25.00	57	75.00	0.599	40	52.63	32	42.11	4	5.26	0.196
G4	30	28.30	9	30.00	21	70.00		21	70.00	7	23.33	2	6.67	
**Platinum sensitivity**														
Highly sensitive (DFS > 24 mths)	22	20.37	6	27.27	16	72.73	0.815	14	63.64	6	27.27	2	9.09	0.342
Moderately sensitive (24 mths> DFS > 6 mths)	42	38.89	12	28.57	30	71.43		21	50.00	20	47.62	1	2.38	
Resistant (DFS < 6 mths)	44	40.74	10	22.73	34	77.27		26	59.09	14	31.82	4	9.09	
**TP53 Accumulation**														
-	40	37.04	8	20.00	32	80.00	0.281	21	52.50	16	40.00	3	7.50	0.806
+	68	62.96	20	29.41	48	70.59		40	58.82	24	35.29	4	5.88	
**CHT response**														
CR	74	68.52	19	25.68	55	74.32	0.546	41	55.41	30	40.54	3	4.05	0.446
PR	31	28.70	9	29.03	22	70.97		18	58.06	9	29.03	4	12.90	
NC or P	3	2.78	0	0.00	3	100.00		2	66.67	1	33.33	0	0.00	
**Growth type**														
Papillary	17	15.74	2	11.76	15	88.24	0.345	9	52.94	4	23.53	4	23.53	0.022
Solid	34	31.48	10	29.41	24	70.59		17	50.00	15	44.12	2	5.88	
Mixed	57	52.78	16	28.07	41	71.93		35	61.40	21	36.84	1	1.75	
**Mitotic activity**														
1 (0-9/10HPF)	11	10.19	2	18.18	9	81.82	0.816	5	45.45	5	45.45	1	9.09	0.396
2 (10-24/10HPF)	25	23.15	7	28.00	18	72.00		11	44.00	11	44.00	3	12.00	
3 (> 24/10HPF)	72	66.67	19	26.39	53	73.61		45	62.50	24	33.33	3	4.17	

Characteristics	Number of patients		POSTN expression in cancer cells				P value	POSTN expression in tumor stroma						P value
			weak (n=28, 25.9%)		strong (n=80, 74%)			weak (n=61, 56.5%)		moderate (n=40, 37%)		strong (n=7, 6.5%)		
	n	%	n	%	n	%		n	%	n	%	n	%	
**Source of tumor sample**														
**Ovary (O)**	39	36.11	8	20.51	31	79.49	0.459	25	64.10	12	30.77	2	5.13	0.138
**Tumor (T)**	33	30.56	11	33.33	22	66.67		22	66.67	9	27.27	2	6.06	
**Peritoneum (P)**	36	33.33	9	25.00	27	75.00		14	38.89	19	52.78	3	8.33	
**O + T**	72	66.67	19	26.39	53	73.61	0.877	47	65.28	21	29.17	4	5.56	0.032
**P**	36	33.33	9	25.00	27	75.00		14	38.89	19	52.78	3	8.33	
**Inflammatory infiltration**														
**Weak**	81	75.00	20	24.69	61	75.31	0.612	44	54.32	30	37.04	7	8.64	0.272
**Moderate + Strong**	27	25.00	8	29.63	19	70.37		17	62.96	10	37.04	0	0.00	
**Degree of desmoplastic reaction**														
**1**	51	47.22	19	37.25	32	62.75	0.037	35	68.63	15	29.41	1	1.96	< 0.001
**2**	47	43.52	7	14.89	40	85.11		25	53.19	20	42.55	2	4.26	
**3**	10	9.26	2	20.00	8	80.00		1	10.00	5	50.00	4	40.00	
**Necrosis**														
**-**	28	25.93	5	17.86	23	82.14	0.258	19	67.86	7	25.00	2	7.14	0.306
**+**	80	74.07	23	28.75	57	71.25		42	52.50	33	41.25	5	6.25	
**Calcification**														
**-**	78	72.22	25	32.05	53	67.95	0.019	46	58.97	28	35.90	4	5.13	0.548
**+**	30	27.78	3	10.00	27	90.00		15	50.00	12	40.00	3	10.00	

*** G2 ignored, because of only two patients with G2;**
HPF – high power field (400-fold magnification);
CHT response: CR – complete response, PR – partial response, NC – no change, P – progression;
O – tumor section containing ovarian structure(s), T – section with tumor tissue only, P – section containing peritoneal/omental structures;
Stromal periostin expression: weak – score 1, moderate – score 2, strong – score 3

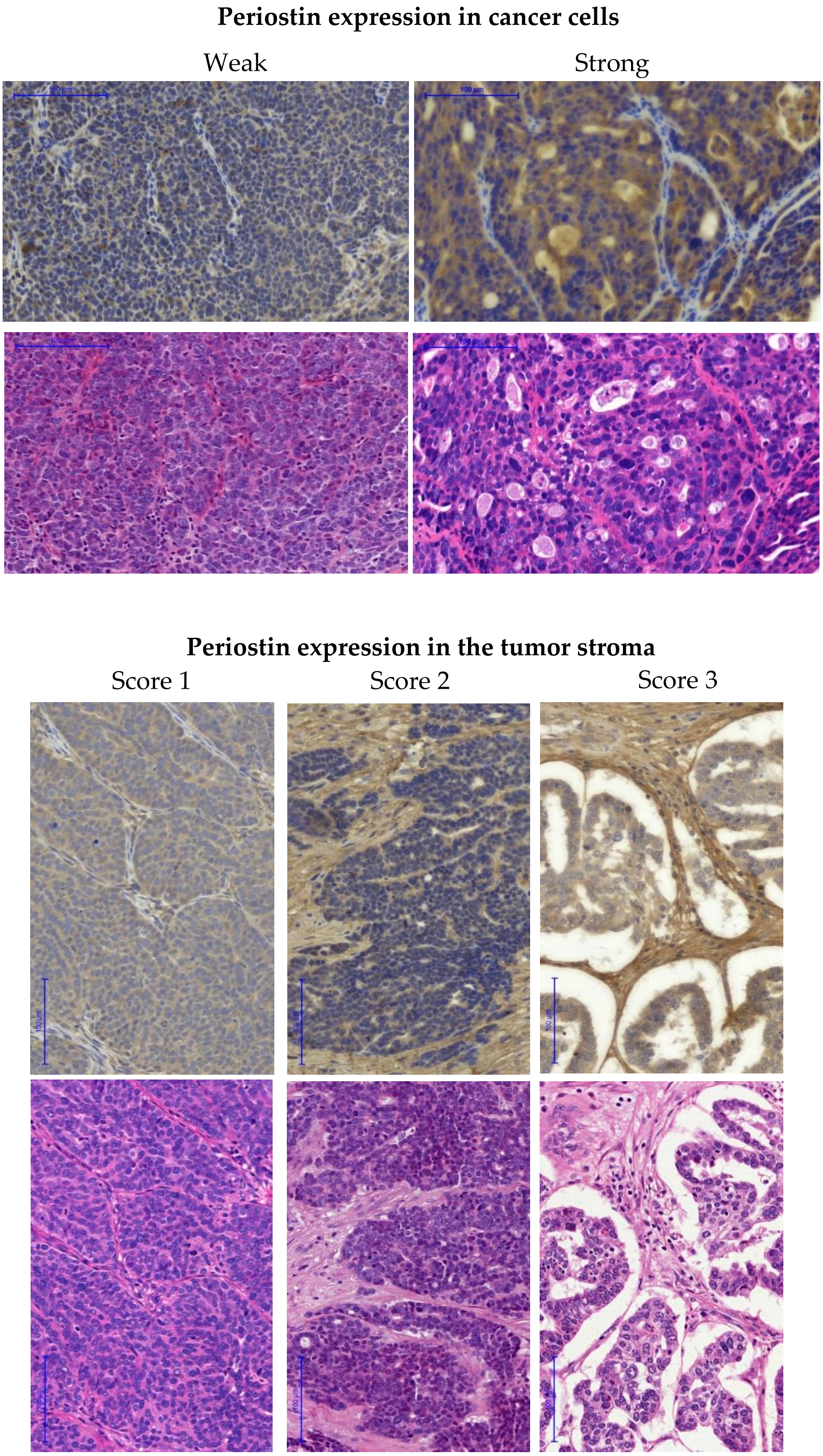

Figure S3. Immunohistochemical detection of periostin. Periostin expression was evaluated separately in cancer cells (upper panel) and in the tumor stroma (lower panel). Images show IHC staining in cancer cells scored either as "weak" or "strong" (below are shown corresponding H&E images), and IHC staining of stromal periostin scored 1 – 3 (below are shown corresponding H&E images). Pannoramic 250 Flash II Scanner, scale bar: 100 μm.

Overall survival

Disease-free survival

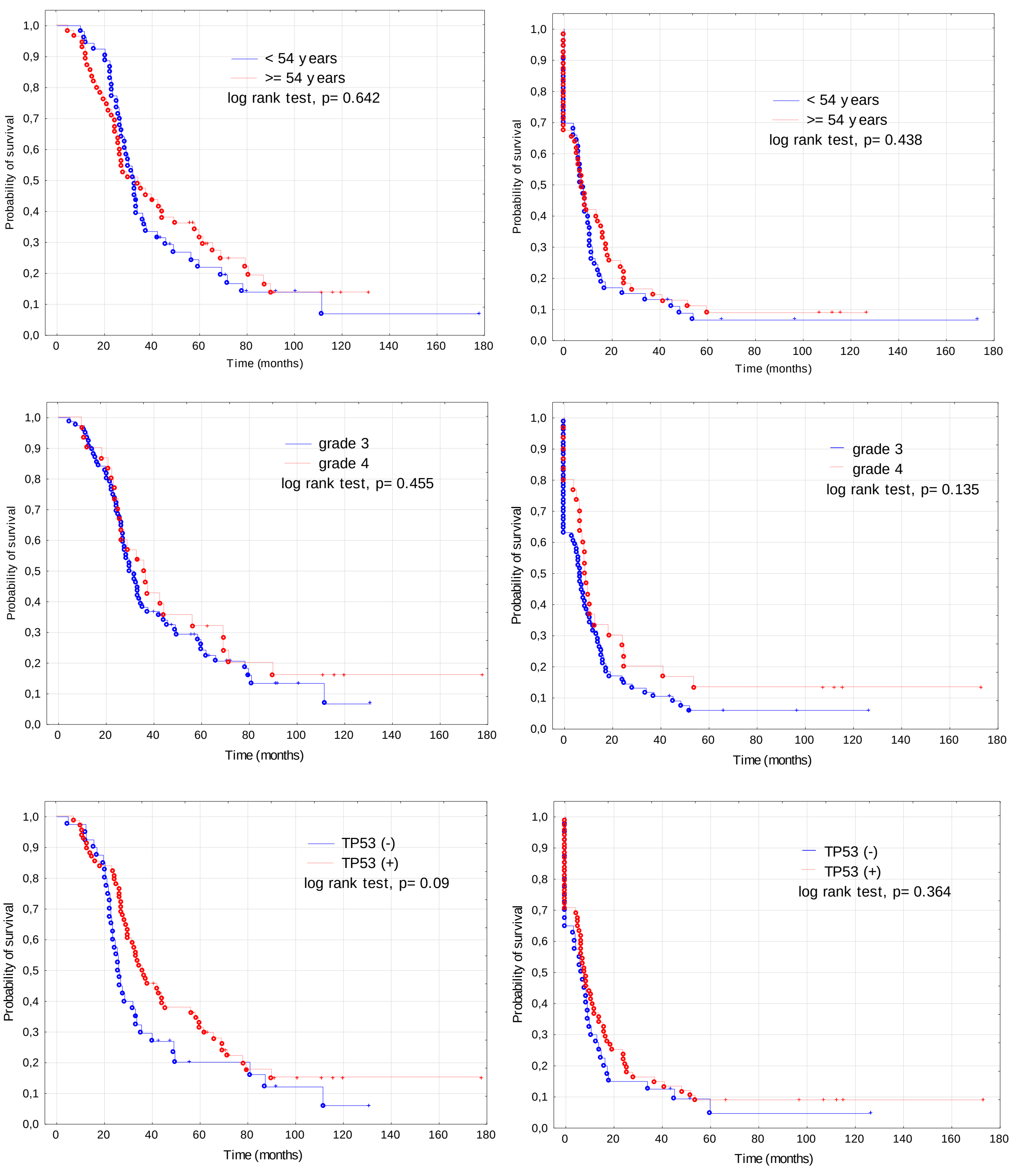

Figure S4. Kaplan-Meier analysis of overall survival (OS) and disease-free survival (DFS) in 108 patients in regard to following clinico-pathological features: age, grade, TP53 accumulation (all nonsignificant).

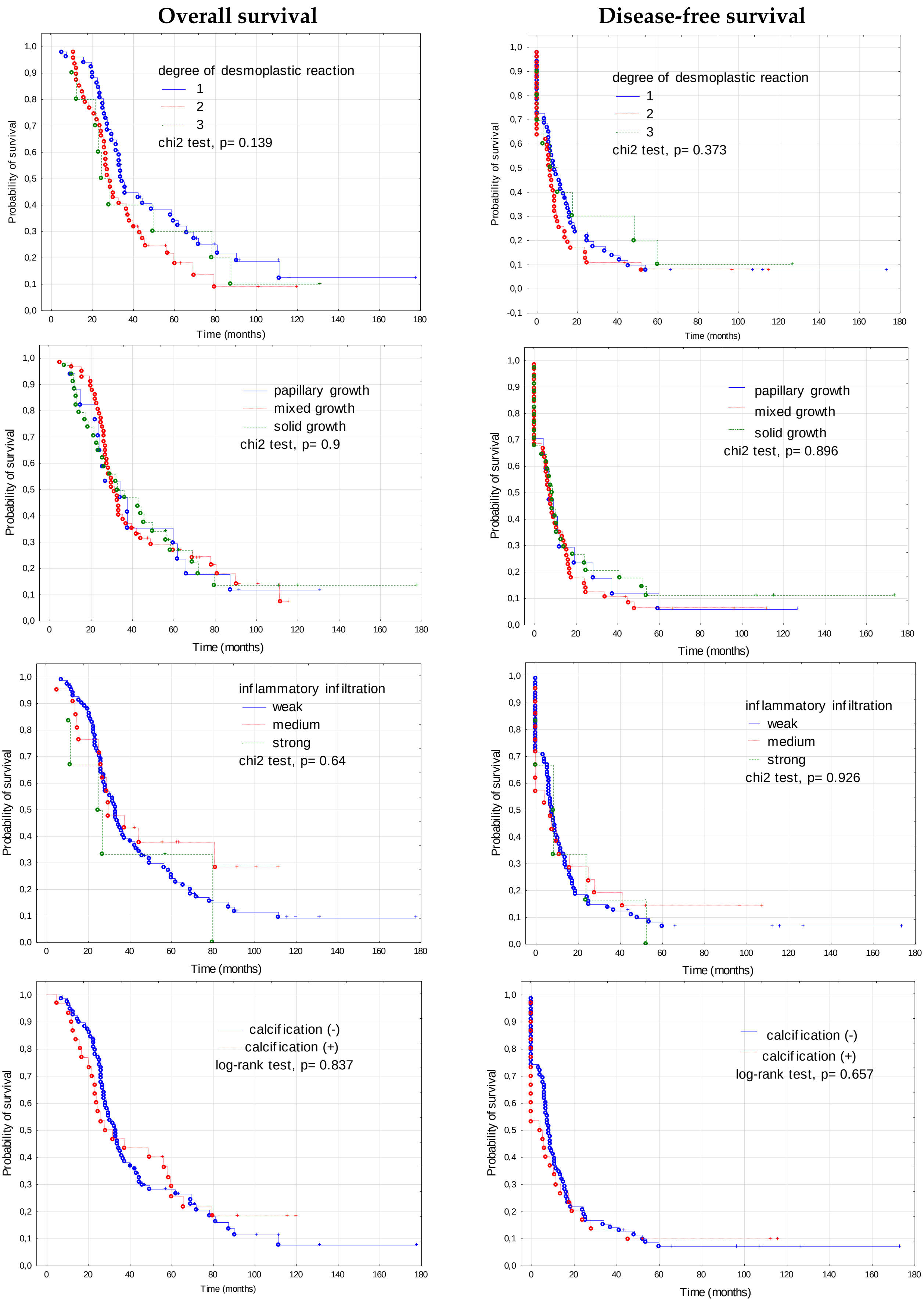

Figure S4 (cont.). Kaplan-Meier analysis of overall survival (OS) and disease-free survival (DFS) in 108 patients in regard to following clinico-pathological features: degree of desmoplastic reaction, type of tumor growth (papillary versus solid versus mixed), inflammatory infiltration, calcifications in the tumor (all nonsignificant).

Stromal FN1	Stromal POSTN	FN1&POSTN score	No. of samples	Median OS months
**1**	1	**2**	14	55.88 (range 4.8-177.83) After exclusion of the outlier: range 32.93-177.83
**1**	2	**3**	2	30.92 (range 10.7-119.93)
**2**	1		42	
**2**	2	**4**	21	28.77 (range 7.3-100.83)
**1**	3		0	
**3**	1		5	
**2**	3	**5**	1	26.55 (range 11.53-91.53)
**3**	2		17	
**3**	3	**6**	6	42.23 (range 9.87-131.17) After exclusion of outlier: range 9.87-87.27

Stromal FN1	Stromal POSTN	FN1&POSTN score	No. of samples	Median OS months
**1**	1	**2**	14	55.88 (range 4.8-177.83) After exclusion of outlier: range 32.93-177.83
**1**	2	**3**	2	30.92 (range 10.7-119.93)
**2**	1		42	
**2**	2	**4-6**	21	27.63 (range 7.3-131.17)
**1**	3		0	
**3**	1		5	
**2**	3		1	
**3**	2		17	
**3**	3		6	

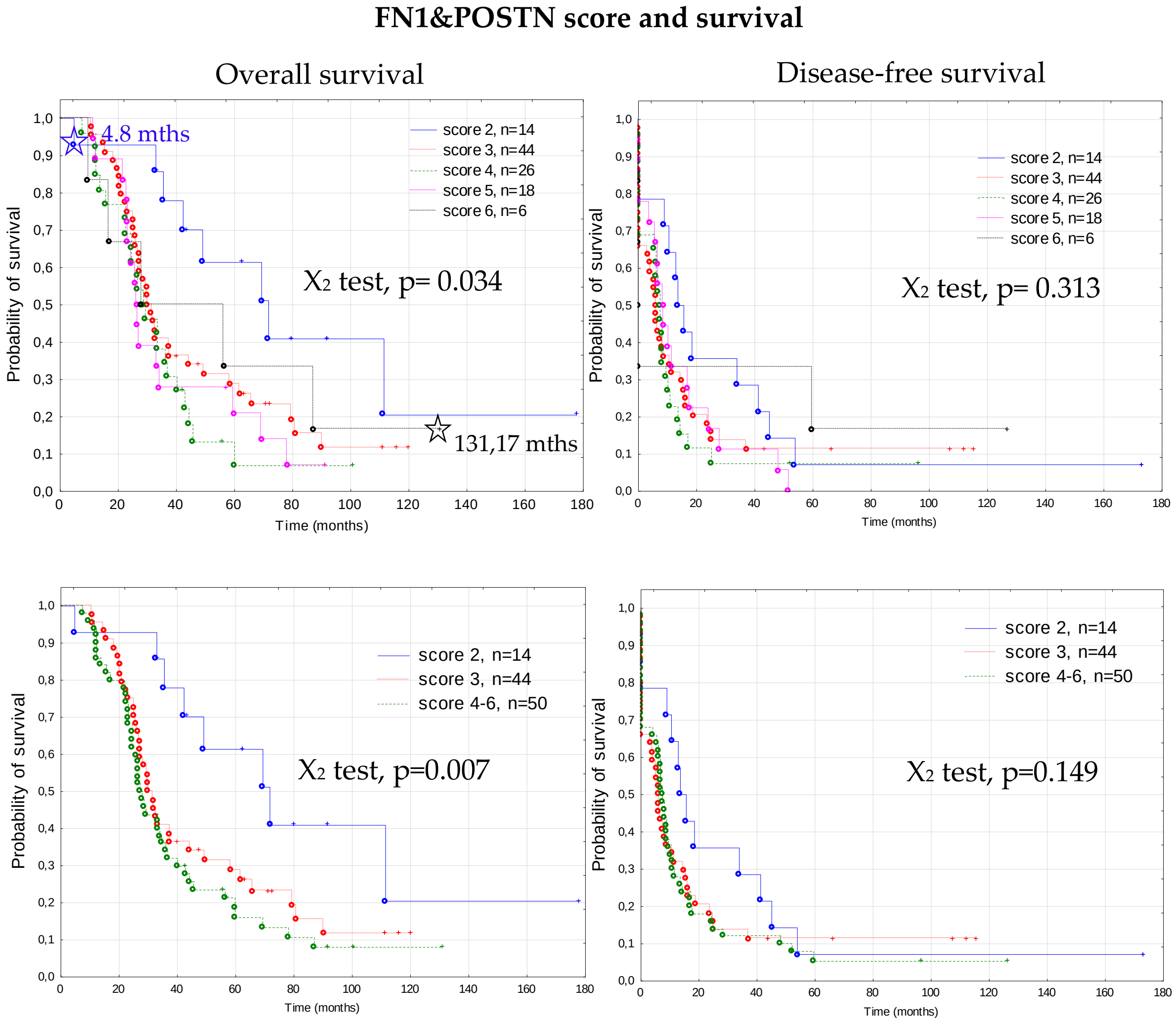

Figure S5. Kaplan-Meier analysis of overall survival (OS) and disease-free survival (DFS) in 108 patients stratified by combined FN1&POSTN score.

Upper panel: 5 groups of patients (score 2, 3, 4, 5, and 6, respectively). Blue asterisk indicates death of the protruding patient with score 2 (best prognosis), who had the shortest OS (4.8 months) from the whole cohort of 108 patients. Black asterisk indicates the protruding patient with score 6 tumor (worst prognosis) who had second longest OS (131,17 months, patient alive) in the whole cohort.

Lower panel: patients with score 2 tumors versus patients with score 3 tumors versus all remaining patients (score 3, 4, 5 and 6 together).

Stromal FN1	Stromal POSTN	FN1&POSTN score	No. of samples	Median OS months
**1**	1	**2**	14	55.88 (range 4.8-177.83) After exclusion of outlier: range 32.93-177.83
**1**	2	**3-6**	2	29.07 (range 7.3-131.17)
**2**	1		42	
**2**	2		21	
**1**	3		0	
**3**	1		5	
**2**	3		1	
**3**	2		17	
**3**	3		6	

FN1&POSTN score and survival

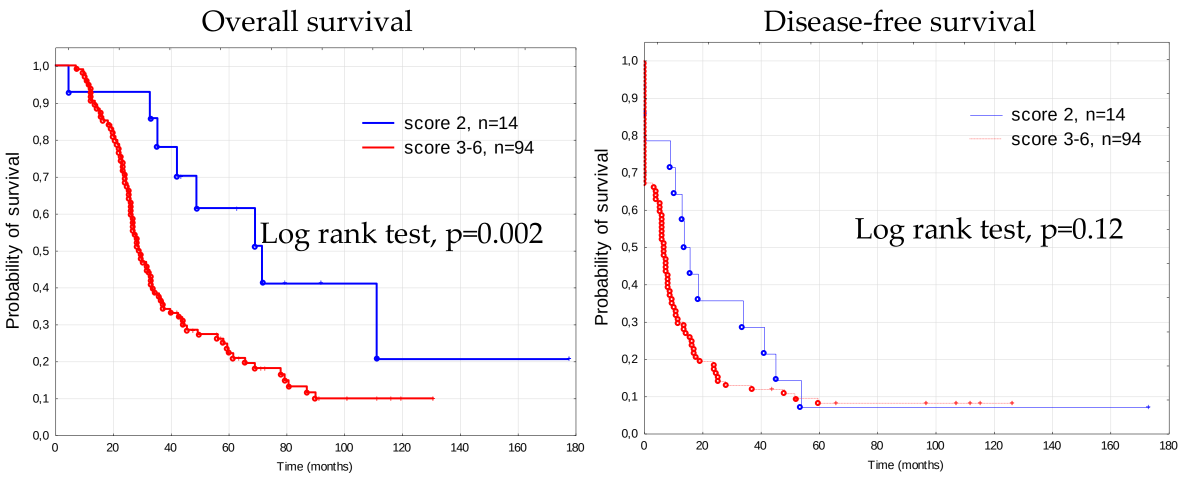

Figure S5 (cont.). Kaplan-Meier analysis of overall survival (OS) and disease-free survival (DFS) in 108 patients stratified by combined FN1&POSTN score: patients with score 2 tumors versus all remaining patients (score 3, 4, 5 and 6 together).
